# A Rare Case of Vomiting-Induced Retrobulbar Hemorrhage

**DOI:** 10.7759/cureus.34839

**Published:** 2023-02-10

**Authors:** Savannah Kumar, Tatyana Beketova, Pearl S Rosenbaum, Abha Amin

**Affiliations:** 1 Medicine, New York Medical College, Valhalla, USA; 2 Department of Ophthalmology, Westchester Medical Center/New York Medical College, Valhalla, USA; 3 Department of Ophthalmology/Ophthalmic Pathology and Oncology, Westchester Medical Center/New York Medical College, Valhalla, USA; 4 Department of Ophthalmology/Cornea, Refractive Surgery and Glaucoma, Westchester Medical Center/New York Medical College, Valhalla, USA

**Keywords:** subconjunctival hemorrhage, valsalva, trauma, ophthalmology, retrobulbar hemorrhage

## Abstract

Retrobulbar hemorrhage may result in sudden accumulation of blood in the retrobulbar space which can lead to an orbital compartment syndrome. This potentially blinding condition is characterized by a rapid increase in intra-orbital pressure. While most commonly associated with orbital trauma, it may rarely occur with Valsalva events in patients on anticoagulants. In this report, we present a case of a retrobulbar hemorrhage secondary to self-induced vomiting, occurring in a patient on no anticoagulation medication.

## Introduction

Retrobulbar hemorrhage (RBH) causing an orbital compartment syndrome can be sight threatening and must be promptly reversed to avoid permanent vision loss due to ischemia of the optic nerve and retina. Symptoms of RBH include severe pain, pressure, loss of vision, diplopia, nausea, and vomiting. Clinical findings of RBH include expanding proptosis, ophthalmoplegia, increased intraocular pressure, loss of pupillary reflexes, and optic disc or retinal pallor [1]. Treatment varies based on visual acuity at presentation and can range from lateral canthotomy and cantholysis in severe, sight-threatening cases to conservative management using digital ocular massage and head elevation [1].

RBH is often associated with orbital trauma and, rarely, with Valsalva events in patients on anticoagulants [2]. In a Valsalva maneuver, the increased intra-abdominal and intra-thoracic pressure results in congestion and rupture of the orbital veins while the concurrent use of anticoagulants predisposes to hemorrhage [3]. In this report, we present a rare case of RBH in a patient with no antecedent trauma and no history of systemic medication use.

## Case presentation

A previously healthy 47-year-old Hispanic woman presented to an outside hospital (OSH) complaining of right eye pain and swelling that started after a prolonged episode of self-induced vomiting. The patient pressed on her uvula shortly after ingesting a large meal; the vomiting was protracted and the patient was unable to terminate it herself. She denied any recent trauma or injuries to the face, head or neck. The past medical and ocular history was significant only for glaucoma, treated topically with bimatoprost.

**Figure 1:**
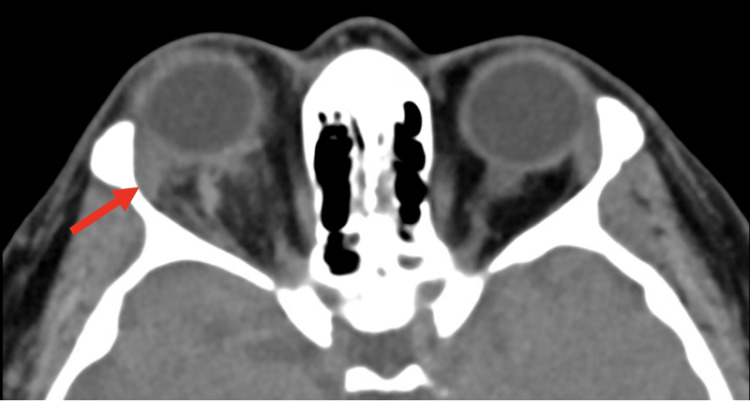
Axial non-contrast CT image showing a hyperdense retrobulbar infiltration and proptosis of the right eye (red arrow)

Non-contrast computerized tomography (CT) of the brain and orbits at the OSH showed right-sided proptosis of 3.98mm and retrobulbar infiltration. The optic nerve sheath complexes were symmetric and unremarkable. The globes were intact bilaterally and the skull base was normal (Figure 1).

**Figure 2:**
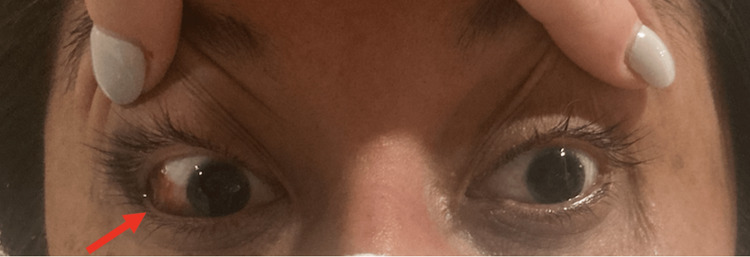
External photograph demonstrating subconjunctival hemorrhage of the right eye (red arrow)

The patient was transferred to our tertiary care hospital for ophthalmologic consultation where uncorrected acuities of 20/25 on the right and 20/20 on the left were obtained. Intraocular pressures measured 21 and 20 mmHg on the right and left, respectively. Pupils were round and reactive bilaterally without an afferent pupillary defect. Color vision, extraocular motility, and confrontational field testing were normal. On the right, mild periorbital edema, mild proptosis, and subconjunctival hemorrhage temporally were noted (Figure 2). External examination of the left eye was normal. The cornea, anterior chamber, lens and vitreous were within normal limits bilaterally. Dilated fundoscopy of both eyes was significant for sharp, pink optic disc with a cup-to-disc ratio of 0.8; the vessels, maculas and periphery were normal bilaterally. A coagulation profile and platelet count were within normal limits. 

A diagnosis of right-sided subconjunctival hemorrhage and RBH was made. As the RBH was not sight threatening, the patient was instructed to utilize digital ocular massage at home and was educated about the risks of self-induced vomiting and Valsalva maneuvers. She was instructed to avoid aspirin, non-steroidal anti-inflammatory medications, heavy lifting and strenuous activity over the next two weeks to avoid hemorrhage expansion and was advised to return to the emergency department if she experienced worsening vision, pain or swelling of the right eye. An outpatient follow-up visit with her local ophthalmologist was also scheduled to address additional ophthalmic concerns and continuation of her glaucoma treatment.

## Discussion

RBH is a rare condition that is most associated with accidental trauma or eyelid or orbital surgery [4]. It is less commonly associated with Valsalva maneuvers in patients on anticoagulants [5]. The Valsalva maneuver is postulated to cause RBH via increased intraabdominal and intrathoracic pressure which results in congestion and rupture of the orbital veins into the retrobulbar space [6].

Diagnosis of RBH typically utilizes ophthalmic examination in addition to CT of the orbits in axial and coronal views to visualize the presence of blood in the retrobulbar space. If ophthalmic examination shows concerning signs of proptosis, vision loss or afferent pupillary defect prior to imaging, the option to forego imaging and proceed with treatment is made with the understanding that the patient likely has an expanding and unstable RBH threatening vision [4]. 

RBH can be treated conservatively with digital ocular massage if non-sight threatening like in the presented case [7]. When RBH is sight threatening, it is treated with a combination of canthotomy and cantholysis in order to decompress the orbit. It is recommended that surgical intervention take place within two hours in order to protect the retina from retinal nerve fiber death and subsequent permanent vision loss [4].

We found this case to be novel and the first reported instance of RBH secondary to vomiting in a patient not on anticoagulant medications. A literature search of PubMed and ScienceDirect using the terms “Retrobulbar Hemorrhage” OR “Retrobulbar Hematoma” AND “Valsalva” OR “Valsalva Maneuver” AND “Vomiting” did not yield any manuscripts describing a case of this nature between 1997 and 2022.

## Conclusions

RBH is a sight-threatening condition typically associated with orbital trauma or orbital surgery. RBH secondary to Valsalva maneuvers is uncommon in atraumatic patients who are not on anticoagulant treatment. Herein we describe the etiology, symptoms, diagnosis and treatment of RBH as well as the first reported case of RBH resulting from self-induced vomiting in a healthy patient on no anticoagulation treatment.

This first-of-its-kind case report highlights the importance of ruling out sight-threatening RBH in patients who present with ocular symptoms following continuous Valsalva maneuvers such as vomiting. 
